# Oncolytic Virus Therapy in a New Era of Immunotherapy, Enhanced by Combination with Existing Anticancer Therapies: Turn up the Heat!

**DOI:** 10.7150/jca.102285

**Published:** 2025-02-18

**Authors:** Emily Charlotte Fretwell, Annwyne Houldsworth

**Affiliations:** University of Exeter Medical School, Faculty of Health and Life Sciences, Exeter, EX2 4TH, UK.

**Keywords:** oncolytic viruses, cancer, T-VEC, metastasis, oncoimmunology, combination therapies, CART-T-cells, cytokines

## Abstract

Oncolytic viral therapy is a promising treatment for cancer, where 'cold' tumour cells can become 'hot' to the host immune system. However, with few FDA approved therapies, development of new strategies for more cancer types has been slow and relatively unsuccessful in recent years, Combination therapy has been successful for other types of cancer treatment, therefore, may be a viable alternative to improve the efficacy of oncolytic viral therapy which may reduce some of the adverse events of currently used monotherapies, oncolytic virus therapy and chemotherapy being mutually complimentary with each other. Combining oncolytic viruses with immune checkpoint inhibitors provides a significant increase in efficacy when viral therapy was combined with the drug ipilimumab.

Phase I and II studies concluded that combination with chemotherapies was safe and effective but did not significantly improve on current monotherapies. Recent experiments suggest that a combination of CAR-T and CAR--M cells is a promising therapeutic approach but needs to advance to clinical testing to observe the human response to the therapy. Viral combination with ipilimumab showed the highest potential for a successful treatment and clinical trials should be advanced to phase III to find conclusive supporting evidence. This review aims to identify and evaluate the potential of currently evolving oncolytic viral therapy with recent advances in genetic engineering providing enhanced oncolytic activity in the tumour, and addressing the lack of host immune responses in 'cold' tumours, with an additional role in enhancing conventional treatment efficacy with combination therapies. The potential of oncolytic viruses to 'turn up the heat' of a tumour microenvironment immunogenicity in combination with other anticancer treatments, provides a promising future for new cancer therapies.

## Introduction

Globally, according to data from Cancer Research UK, there were 17 million new cases of cancer and 9.6 million deaths recorded in 2018 with this predicted to rise to 27.5 million new cases per year by 2040. 1 Only 50% of patients diagnosed with cancer survive for 10 or more years after diagnosis suggesting a need for the development of alternative therapies that deviate from the classical treatment options of surgical resection, radiotherapy, targeted drug therapy and chemotherapy. 1,2 In recent years, there has been an increase in research into the potential of using immunotherapies for the treatment of cancers including the use of oncolytic viruses (OVs).

The concept of genetic engineering to transform viruses into vectors that express anti-tumour factors is explored and compared with more conventional chemotherapy, focusing on transformed oncolytic viruses (OVs) that can enhance the immunogenicity of tumours to attract the host immune system by 'heating up' the tumour and its environment, in terms of inflammatory processes. Combination therapies with chemotherapy and immunotherapy are discussed with existing and possible genetic engineering to transform OVs to express other anticancer treatments. Recent animal studies and their translation to clinical trials aim to identify and evaluate the potential of combining oncolytic viral therapy with currently approved cancer therapies, considering how they may be mutually complimentary to each other, and where the efficacy of a monotreatment can be improved.

Examples include genetic transfection of cytokine genes, like IL-12, and factors that stimulate the immune responses to tumours. Capsid modifications can alter the tropism of some viruses enabling them to target specific tumours. 3,4 Strategies to combine approved cancer therapies with OVs and immunotherapies with ways to remodel the tumour microenvironment (TIME) to be more immunogenic to the host's immune system are also discussed.

## Oncogenesis

Unlike normal cells, cancer cells have multiple nucleoli, and a small cytoplasmic volume and grow uncontrollably causing hyperplasia, dysplasia, and neoplasia of immature cells. Undifferentiated cells described as anaplasia result in morphological changes to cells and these can have genetic mutational changes such as tumour suppressor genes, like P53 or Rb genes. Indeed at least six genetic changes are recognised in cancer mutagenesis known as proto-oncogenes, for example, immune checkpoint inhibitor (ICI) genes and factors that inhibit apoptosis, to be discussed later. Oncogenic mutations and epigenetic aberrations accumulate progressively in the carcinogenesis process. These changes can result from several targets that can be used for immunotherapy. 5

Rapidly proliferating and replication of cancer cells means that they can be targeted by chemotherapy by targeting microtubules, however, this also targets normal cells.

Some oncogenic genes drive angiogenesis by expressing pro-angiogenic factors, like, vascular endothelial growth factor (VEGF) that promote neo-vasculature to the tumour, however, without a sufficient supply of blood, a tumour can become necrotic.^6^

Another feature of oncogenesis is the evasion of the host immune defence where cancer cells exhibit antigenicity and immunogenicity. Malignant cells acquire immunosuppressant properties, including the expression of suppressive cytokines like IL-10 and TNF-β and intrinsic immune resistance is common. 7

## Epigenetics and cancer

The phenotype of cancer cells often differs from that of normal cells due to epigenetic changes in genes, this leads to altered gene function and cellular transformations that occur during malignant changes. Cancer epigenetics reprogramming happens due to histone modifications, nucleosome positioning, DNA methylation and non-coding RNAs. Reversing these transformed epigenetic landscapes is an innovative area for the potential design of new treatment strategies.^8^ DNA methyltransferases (DNMTs) are potential therapeutic target enzymes to reverse the methylation of genes as they regulate the process of DNA methylation and can be used as a biomarker for tumorigenesis. 9 An example of this strategy is oral azacytidine therapy as an epigenetic modifier. 9 Entinostat has antineoplastic properties and is another histone deacetylase (HDAC) inhibitor that promotes the activation of some gene transcription by promoting histone acetylation.

Ubiquitylation is posttranslational attachment of ubiquitin to proteins and some members of the ubiquitin family are found to be dysregulated in cancer, being amplified in some cancers. Members of the deubiquitylating enzyme family that remove posttranslational modifying ubiquitin are considered potential anticancer drugs.^10^

Hypomethylating agents such as azacytidine, a chemotherapy drug, reduce the effects of dysregulated gene expression through epigenetic changes used for leukaemia-type cancers, particularly myelodysplastic syndromes. 11

## Cold and hot tumours

A 'cold tumour' is identified by different immune mechanisms and the tumour immune microenvironment (TIME), including the degree of lymphocyte infiltration and immunosuppressive pathways of immune checkpoints. 12 Thus, cold and hot tumours also exhibit different signalling mechanisms, such as programmed death receptor-1 PD-1), programmed death receptor-1 ligand (PD-L1), cytotoxic T-lymphocyte antigen-4 (CTLA-4), T-cell receptor (TCR) and major histocompatibility factor (MHC) as some examples. A 'hot' tumour has enhanced immunogenicity by being inflamed and infiltrated by T-cells activated against factors, such as preexisting antitumor immune responses and potential genomic instability. A 'hot' or 'cold' tumour can be identified by its cytotoxic T-cell status within the tumour with the number of T-cells and natural killer (NK) cells present.^12^,^13^

Examples of 'cold tumours' that do not respond to immunotherapy nor trigger an immune response from the host immune system include, breast, ovary, prostate, pancreas and glioblastoma. Some breast cancers are described as triple negative as they do not express progesterone or oestrogen receptors and lack expression of HER2. [14-16]About 10-20% of this type of breast tumours are triple negative when they are diagnosed. Modulation of the cancer immunity cycle of a cold immunosuppressive tumour can undergo immunomodulation into a 'hot' tumour and this can be achieved by a number of methods.

There are currently 62 clinical trials based on OVs that make tumours more immunogenic in breast cancer patients. 17 An example of the transformation of a 'cold' tumour to a 'hot' tumour is a genetically engineered trial of CF33-hNIS-antiPDL1, as the first human clinical trial (NCT05081492) of an OV combined with a checkpoint inhibitor to PD-L1 is currently being trialled as a treatment for metastatic breast cancer. 18,19

A recent publication reports that an oncolytic herpes simplex virus type-1 vaccine strain (VC2) can improve tumour T cell infiltration of stage four breast cancer cells in a 5T1/Balb/c mouse model. The primary tumour was not reduced significantly but lung metastases were significantly reduced. The lung metastases were infiltrated with CD4+ and CD4+CD8+ double-positive T cells and presented a significant improvement in immune responses to cancer compared to controls. 20 This improvement of T-cell response against the tumours with a reduction of PD-L1 and vascular endothelial growth factor (VEGF) expression is an example of inducing a 'hot' tumour when compared to the lack of immune infiltration in 'cold' breast tumours or the controls in this experiment. 21 The quantity of tumour infiltrating lymphocytes is considered to be a good predictive biomarker of therapy responses, where intratumoral CD8+ is considered to have a better prognosis. The hotter the tumour, the more tumour infiltrating lymphocytes present. 22

An oncolytic measles virotherapy (rMeV-Hu191) also shows promising multifaceted anti-tumour responses in breast cancer xenograft mouse models. 23

Another example of 'turning up the heat' is the treatment of melanoma, where immune checkpoint inhibitors and oncolytic viruses have been successfully employed. T-VEC (Imlygic) is injected directly into the tumour where it infects and kills melanoma cells while alerting the host immune system to new epitopes. 14,15,24

CD73, ecto-5′-nucleotidase, is an enzyme encoded by the NT5E gene responsible for generating immune suppressive adenosine. This is more highly expressed in tumours thus making the tumour 'colder' to the host immune system.^25^ There is some promise in cancer immunotherapy preclinical trials using Inhibitors of the CD73 adenosinergic checkpoints. 26

## Immune checkpoint inhibitors

ICIs are being increasingly studied that can alter the landscape of the TIME by reversing T-cell exhaustion and the reinvigoration of anti-tumour activated T-cells. By 2021, eight drugs had been approved for the treatment of numerous cancers. 27

ICI involves the use of antibodies to block receptors such as PD-1 and CTLA-4; receptors utilised by cancer cells to dampen the immune response by inhibiting the activation of T-cells creating an immunosuppressant environment. 27 Cancer cells escape apoptosis via this interaction of PD-1 and CTLA-4 with T-cells but monoclonal antibodies to these molecules can induce tumour cell death. PD-1 inhibitor, nivolumab, and CTLA-4 inhibitor, ipilimumab, have been tested in combination with one another resulting in longer PFS in melanoma patients, however, caused a greater proportion of adverse events. 27,28 Anti-PD-1 is safe to administer to patients, even with cardiac, hepatic and renal dysfunction. 29,30

It is suggested that OVs may have the potential to increase the efficacy of ICI when used concurrently, possibly with fewer negative side effects, and may allow ICI to be used for a wider range of patients. Some 'cold' tumours are poorly immunogenic with low mutational load, or PD-L1 and MHC-1 expression. Enhanced tumour cytotoxicity interactions, making the tumour 'hot' and inflamed, may be achieved by combining two or more types of therapy, enhancing the immunogenicity of the TIME, increasing PD-L1 expression and activating natural killer cell engagement (Figure 2). 31,32

## Oncolytic viruses

OVs are genetically modified viruses that work to eliminate cancer by infecting tumour cells, whilst avoiding healthy cells, leading to an increase in cytotoxicity towards cancer cells and resulting in cell lysis. 33

Many viruses are being considered as oncolytic therapies, such as polio, HSV, measles, vaccinia, Zika virus and even HIV depending on the receptors required for entry into tumour cells. Some animal viruses have also been found useful as OVs, like vesicular stomatitis and the Newcastle virus that infects pigeons. The tropism of the virus can facilitate entry into specific tissues. 31,34

**Figure 1:**
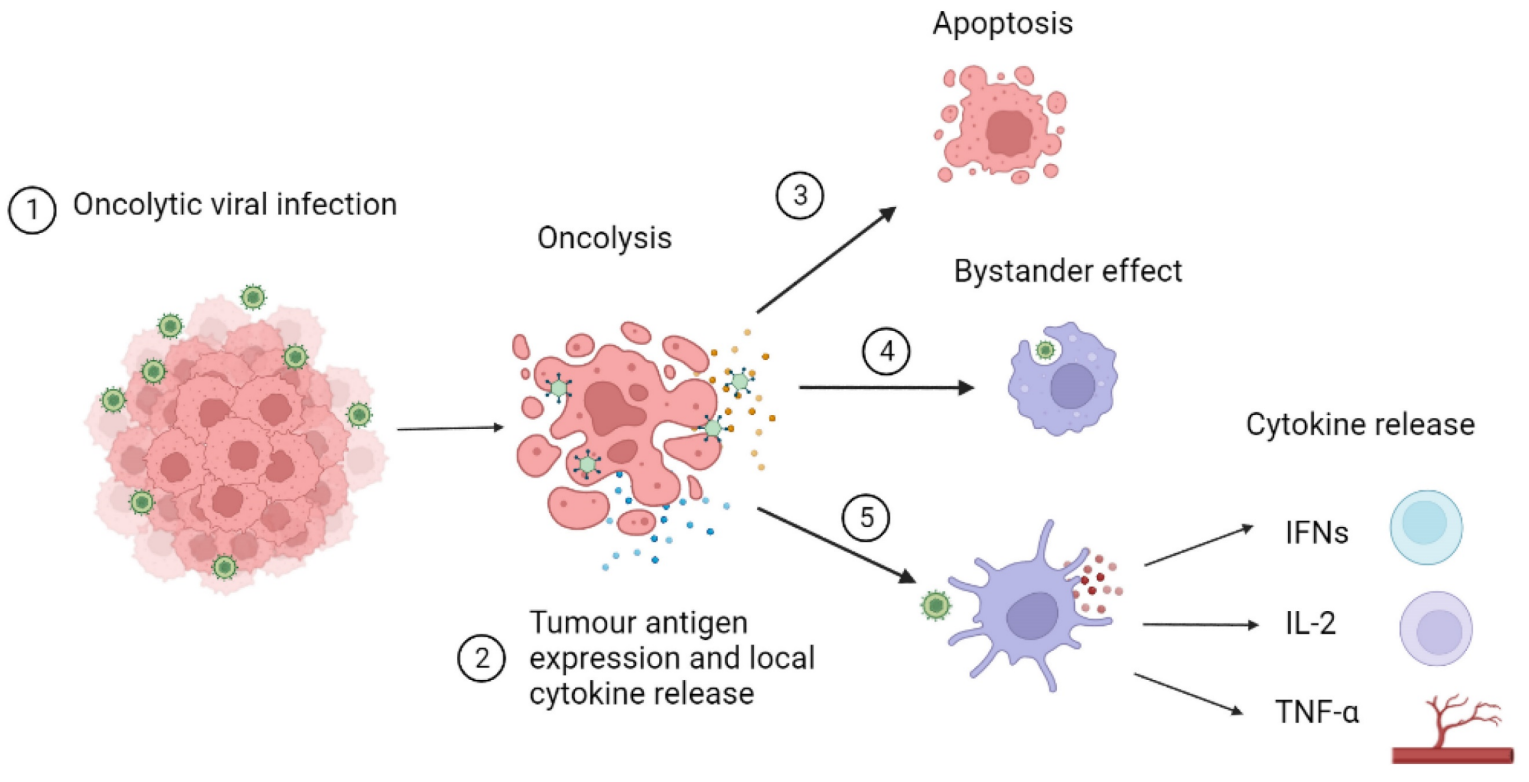
OV (OV) infects tumour cells through recognition of abnormal surface markers such as CD20 and endothelial growth factor receptor. **2)** OV increases tumour antigen expression converting “cold” tumours into “hot” tumours reducing the immune-suppressant environment and increasing recognition by CD8+ T cells. **3)** Tumour cells undergo oncolysis resulting from viral interruption in cellular functions leading to apoptosis and necrosis. **4)** Local release of cytokines activates bystander effect triggering an immune response towards nearby tumour cells. **5)** Dendritic cells recognise pattern associated molecular patterns or viral RNA/DNA and release cytokines and chemokines. Type I interferons (IFN) activate B cells and dendritic cells. Interleukin 2 (IL-2) stimulates the activation of CD8+ and CD4+ T cells, activates natural killer cells and inhibits T regulatory cells. Tumour necrosis factor alpha (TNF-α) stimulates T helper cells and leads to the destruction of blood vessels supplying tumour cells.^5^

An OV infects tumour cells through the recognition of abnormal surface markers such as CD20 and endothelial growth factor receptors. This infection increases tumour antigen expression converting “cold” tumours into “hot” inflammatory tumours by the immune-suppressant TIME and increasing recognition by CD8+ T-cells.^13^,^35^ These CD8+ T-cells infiltrate tumours and can transition into phenotypes that are optimally effective against cancer cells. 36 Tumour cells then undergo oncolysis resulting from viral interruption in cellular functions leading to apoptosis and necrosis. Local release of cytokines activates a bystander effect triggering an immune response towards nearby tumour cells. Dendritic cells recognise pattern-associated molecular patterns or viral RNA/DNA and release cytokines and chemokines. Type I interferons (IFN) activate B cells and dendritic cells. Interleukin 2 (IL-2) stimulates the activation of CD8+ and CD4+ T-cells, activates natural killer cells and inhibits T regulatory cells. Tumour necrosis factor alpha (TNF-α) stimulates T helper cells and leads to the destruction of blood vessels supplying tumour cells. This mechanism of action of OVs is described in more detail in Figure 1. 37-39 The tumours that do not elicit an immune response and are termed to be 'cold', whereas, as previously described, they are known as ''hot' when the host immune system mounts an immune response to the tumour when they sit in an inflammatory immune microenvironment. 13,35

The TIME involves the action of cytokines that regulate the immune response and direct how cells respond and they are highly critical elements in the pathogenesis of cancer. Tumour evolution and growth can be, both positively and negatively influenced by growth factors like TGF-β, VEGF, and EGF as well as cytokines such as interleukins, interferons, tumour necrosis factors, and other chemokines., It is suggested that the effects of these influential molecules and their signalling pathways could be modulated, in combination with OVs as cancer therapies, especially if the TIME can be regulated by engineered cytokine variants, or their receptor inhibitors as well as monoclonal antibodies and bispecific antibodies. 40

The potential of oncolytic viral therapy (OVT) has been discussed since 1904 when observations showed a patient with acute leukaemia went into remission after viral infection; however, over 100 years later, there remains only one FDA-approved OVT. 34 In 2015, the previously mentioned T-VEC induces antitumour immunity in the patient by replicating within the tumour and causing necrosis and cell death of [the tumour cells. 37 This suggests OVs alone may not be the future of cancer treatment as there has been slow progress and development in recent years. Combination therapy can be a successful method to enhance existing therapies and therefore could be exploited for improving OVT efficacy with plenty of clinical trials completed or currently ongoing; some ongoing trials are summarised in Table 1. 38-42 An example of an ongoing phase III trial is OlviVec, a vaccinia virus, combining olvimulogene nanivacirepvec, chemotherapy with antibodies.^43^

## Discussion

### Chemotherapy and other combination therapies

#### Chemotherapy and other combination therapies

Chemotherapy is considered a classical cancer therapy and acts by interrupting cell growth and includes drugs such as carboplatin, gemcitabine and paclitaxel. 43 Unfortunately, most chemotherapy drugs do not discriminate between normal and cancer cells and target any rapidly replicating tissue. 44

Multiple phase I and phase II clinical trials investigating the efficacy of combining various chemotherapy drugs with OVs for the treatment of a range of different cancer types have been undertaken in recent years with varying degrees of success.

All the trials discussed above investigated OVT using the same virus however the majority combined OVT with other chemotherapy drugs and treated different types of cancers with differing grades, stages, and levels of metastasis and mutations. These factors may affect how successful the OV is at eliciting and enhancing the anti-tumour immune response by making the tumour more immunogenic to the host immune system. For example, Cohn *et al.* (2017) suggested that the pelareorep virus might require activated RAS mutations for optimum action however only 20% of ovarian cancers possess these mutations, therefore this virus may not be suitable for every type of cancer.^45^ Also, the pelareorep virus has not yet been FDA-approved for use as an anti-cancer treatment therefore it may have been more logical to use the already-approved, T-VEC virus.

In addition, the two more successful trials mentioned above, Mahalingam *et al.* (2018) and Mahalingam *et al.* (2017) were both single-arm trials and made their comparisons against historical data for mono treatment with the corresponding chemotherapy, compared to the two unsuccessful trials which used their controls alongside the intervention being tested. 46,47 The use of historical controls makes comparison of results more difficult, and potentially less reliable, due to a lack of control over blinding, randomisation and inclusion and exclusion factors; conditions such as drug doses and treatment regimens may differ. A more recent meta-analysis of the effectiveness of pelareorep and chemotherapy showed no significant improvements in OS, PFS, or ORR in advanced solid tumour patients. 48

PD-1 inhibitor, in combination with oncolytic vaccinia virus (JX-594) is deemed a safe and effective option for metastatic renal cell carcinoma, as it reduced the metastatic and primary tumour burden with less damage to the liver in animal models and clinical trials for this combination are currently ongoing.^49^

Recently, the long-term effectiveness of combined OVT has been documented in colorectal cancer patients and the transformation of a 'cold' TIME to a 'hot' one. 50

The combination of T-VEC and ipilimumab (monoclonal antibody to CTLA-4 for the treatment of advanced melanoma has shown signs of success. 34 A phase Ib trial, conducted by Puzanov *et al.* (2016), observed no dose-limiting toxicities and reported a 26.3% incidence of grade 3 or 4 adverse events; similar to ipilimumab monotherapy, indicating combining the two therapies doesn't increase safety concerns. 16,51 Furthermore, they found this combination resulted in an objective response rate (ORR) of 50%, an 18-month PFS of 50% and an OS of 67%; although this study had a small sample size of only 19 patients and used historical controls. [51]Turning cold tumours into hot tumours by improving T-cell infiltration. 51 Nevertheless, Chesney *et al.* (2018) performed a phase II study with 198 participants which supported the previous results. The study reported an ORR of 39%, significantly higher than monotherapy historical controls for both T-VEC and ipilimumab single therapies, however no significant increase in PFS and OS when compared to controls; possibly revealed because of the large difference in sample sizes between the two studies described. 38,51,52

In contrast to the encouraging results from the ipilimumab combination, pembrolizumab and T-VEC combination therapy for advanced melanoma was regarded as less promising. An initial phase Ib trial, by Ribas *et al.* (2017), showed no increase in toxicity with no occurrence of dose-limiting toxicities throughout the trial and an ORR of 62%, significantly higher than historical PD-1 therapy data[.^32^ However, when the same combination was investigated in a randomised, double-blind phase III trial of 692 participants, by Chesney *et al.* (2022), the significant difference in ORR disappeared and no differences were found between PFS and OS; ORR for pembrolizumab T-VEC combination therapy was 48.6% compared to 41.3% for the pembrolizumab placebo group.^53^

Variations in results may be accounted for by the large difference in sample sizes and the use of internal controls in the phase III trial compared to historical controls for the phase Ib trial. There were also differences in the treatment regimens and participant's previous treatment status. Ribas *et al.* (2017) injected their patients intratumorally every two weeks with T-VEC and pembrolizumab. Alternatively, Chesney *et al.* (2022) injected intratumorally every three weeks. 20,46 Participants from Puzanov *et al.* (2016) and Ribas *et al.* (2017) must not have had any prior systemic treatment, apart from adjuvant therapy, before enrolment into the study. However, Chesney *et al.* (2022) stated that participants could have prior systemic therapy if they had a BRAF mutation, potentially contributing to a significant difference in outcomes. 32,38,51,54

### Chimeric Antigen Receptors T-cell/Macrophage (CAR-T/M) cell therapy

Chimeric antigen receptor T-cell (CAR-T) therapy has gained momentum after the FDA approval of a CD19-targeted CAR-T-cell for treating acute lymphoblastic leukaemia in 2017. 55,56 CAR-T-cell therapy involves the modification of T-cell receptors, which allows them to recognize tumour-associated antigens, increase the specificity and fidelity of tumour targeting and alter the TIMETIME to enhance the efficacy of treatments. 55 However, CAR-T-cell therapy has been associated with adverse events such as cytokine storms and pulmonary toxicity and is ineffective in solid tumours compared to tumours of the blood. 56,57 Combination with OVs may allow improved migration of CAR-T-cells to tumour sites and prolong survival in TIME as well as reducing the adverse events. Genetically modifying OVs can introduce transgenes into TIME to enhance immunogenetic cell death by T-cells. 42,58 A preclinical study with OV, rVSV-LCMVG, in combination with adoptively transferred T-cells, induced fewer neutralizing antibodies to the virus.^59^ Recent advances in cancer treatments in this field have greater penetration capability of solid tumours with CAR-M, a personalised therapy from patient monocytes, using macrophages that target the tumour within TIME, with precision. 60

In pre-clinical studies, OVs have demonstrated they can alter the TIME, using cytokine and chemokine production permitting increased infiltration and activation at the tumour site.^56^ Liu *et al.* (2022) studied the combination of herpes simplex virus T7011, engineered to express chemokine CCL5, cytokine IL-12 and anti-PD-1 antibody, alongside CD19 or BCMA-targeted CAR-T-cells in treating a variety of solid tumours.^56^ It was reported that the virus helped to deliver CD19 and BCMA antigens to the cancer cell surface promoting activation and enhancing CAR-T-cell mediated killing and CCL5 increased CAR-T-cell infiltration to the tumour site. 56 The combination therapy showed improved killing of *in vitro* cells from human laryngeal carcinoma, human melanoma and human prostate carcinoma cells, when compared to the normal T-cell control. 56 In support, Nishio *et al.* (2014) identified adenovirus Ad5Δ24 combination with GD2 targeting CAR-T-cells for the treatment of neuroblastoma also resulted in enhanced CAR-T-cell trafficking and survival at tumour sites likely mediated by the release of cytokines RANTES and IL-15. [61]They found combination therapy reduced the volume of residual tumour cells to 5% compared to 33% in the virus and normal T-cell control, and when transferred to an *in vivo* mouse xenograft model, faster lysis of tumour cells and better control over tumour growth was observed. 61

A combination of OVT and CAR-T-cells also shows increased survival in mouse xenograft models. Rosewell Shaw *et al.* (2017) found that mono treatment for head and neck squamous cell carcinoma using HER2-targeted CAR-T-cells led to mice survival for 25 days, however, when this was combined with an engineered oncolytic adenovirus, mice survival was significantly improved to over 100 days. 62 In agreement, Chalise *et al.* (2022), observed a significant increase in mouse survival when herpes virus simplex G47D was combined with picoplatin-targeted CAR-T-cell compared to both monotherapies and T-cell controls. 57

Pre-clinical experiments combining CAR-T-cell and OVs appear to be encouraging, however, Chalise *et al.* (2022), for example, treated artificial cell lines during *in vitro* experiments, but when these were translated into animal xenograft models using patient-derived tumour cells, the efficacy of the therapy declined suggesting there is still work to be done to translate and reproduce the promising results in animal models, especially if they are to be a successful treatment for humans. 57 Alongside this issue, the studies all looked at the effects of viral production of cytokines and immunomodulators however these molecules all differed. Rosewell Shaw *et al.* (2017) focused on cytokines including IL-21 and IL-12p70, Nishio *et al.* (2014) focused on RANTES and IL-15 and Chalise *et al.* (2022) IFN-γ. This makes it difficult to understand whether success is dependent on the specific virus used or if the specific molecules produced are more responsible.

Generally, there are several different methods to improve T-cell priming, activation, expansion, trafficking and infiltration, enabled by the OV, as mechanisms to turn a 'cold' tumour into a 'hot' one. 35

### Pelareorep

Pelareorep, a serotype 3 reovirus, was used as the OVT in multiple phase II studies in combination with current chemotherapies. 45,47,63 When pelareorep OVT was combined with gemcitabine for the treatment of advanced pancreatic adenocarcinoma, Mahalingam *et al.* (2018) concluded that survival rates were higher than when compared to gemcitabine treatment alone, presenting a clinical benefit rate of 58% and a median overall survival (OS) of 10.2 months. 47 They reported that previous phase II studies, investigating gemcitabine monotherapy, stated a similar progression-free survival (PFS) rate to the combined therapy, which had a median PFS of 3.4 months, but combination therapy suggested lower toxicity with fewer adverse events occurring in participants. 47 Pelareorep has also been combined with carboplatin and paclitaxel to treat melanoma; Mahalingam *et al.* (2017) concluded combination therapy resulted in an improvement in PFS and OS when compared to historical controls. However, this trial was terminated before any further progression due to the success of alternative treatments for melanoma such as novel targeted therapy. 46

Alternatively, treatment for ovarian, tubal, and peritoneal cancer was deemed unsuccessful when pelareorep was combined with paclitaxel. Cohn *et al.* (2017) conducted a phase II randomised control trial where participants were given either paclitaxel monotreatment or paclitaxel in combination with pelareorep virus. Results determined there were no significant differences between each trial arm for PFS or OS; PFS was 4.3 months for mono treatment and 4.4 months for combined treatment and OS was 13.1 for mono treatment and 12.6 for combined. 45 A second randomised control trial, Bernstein *et al.* (2018), examining the effects of the same treatment, but for breast cancer, came to similar conclusions showing no significant differences in PFS or OS when baseline circulating tumour cell levels were considered. 63

### Delivery of therapy

An aspect of OV cancer therapy clinical trials that should be considered is the method of delivery. Different types of delivery of treatments have various advantages and disadvantages, depending on the therapy or the type of tumour to be treated. For example, intratumoral inoculation of an OV enhances topical engagement and has a robust inflammatory immune response while maximising the drug concentration, and is employed for sarcoma or melanoma 64-66 Other methods employed are thoracoabdominal, and intravenous injections depending on the types and location of the tumours. Intravenous delivery can be by injection, cannula or catheter. Other targeting techniques include redesigning viruses by engineering the viral capsids to target tumour cell receptors. 67 Also, the transfection of exogenous genes into the OVs can greatly enhance the penetration of the tumour and oncolysis. 60,60 These different modes of delivery can determine the viral spread, resistance and the level of antiviral immunity from the host immune system. 68

As intracellular parasites, different viruses' pathogenesis relies on specific cell receptors for different tissues, for example, polioviruses target nerve cells and HIV targets CD4 T-cells via CCR5. 69 Viruses can be reengineered, and their tropism can be targeted to specific tissue so that their viral capsid targets specific tumour cells, without infecting surrounding normal tissue. 70

Some of the barriers to OV infection of tumours include low availability of tumour receptors and pre-existing antibodies to the oncovirus being used. To overcome these obstacles to infection different vehicles have been developed. Nanoparticles and stem cells have proved beneficial as have microparticles, hydrogels, extracellular vesicles and shielding polymers, liposomes, and albumin as delivery systems, enabling an OV to be administered intravenously rather than topically, which can also decrease the viral virulence as the host immune system may neutralise the OV before it affects the tumour. 71-73

The antibody conjugation of nanoparticles can reduce the toxicity of chemotherapy drugs, improve loading capacity and enhance the targeting of the drugs, which can enhance the delivery of combination therapies, including immunotherapies and OVT. 74,67

**Figure 2:**
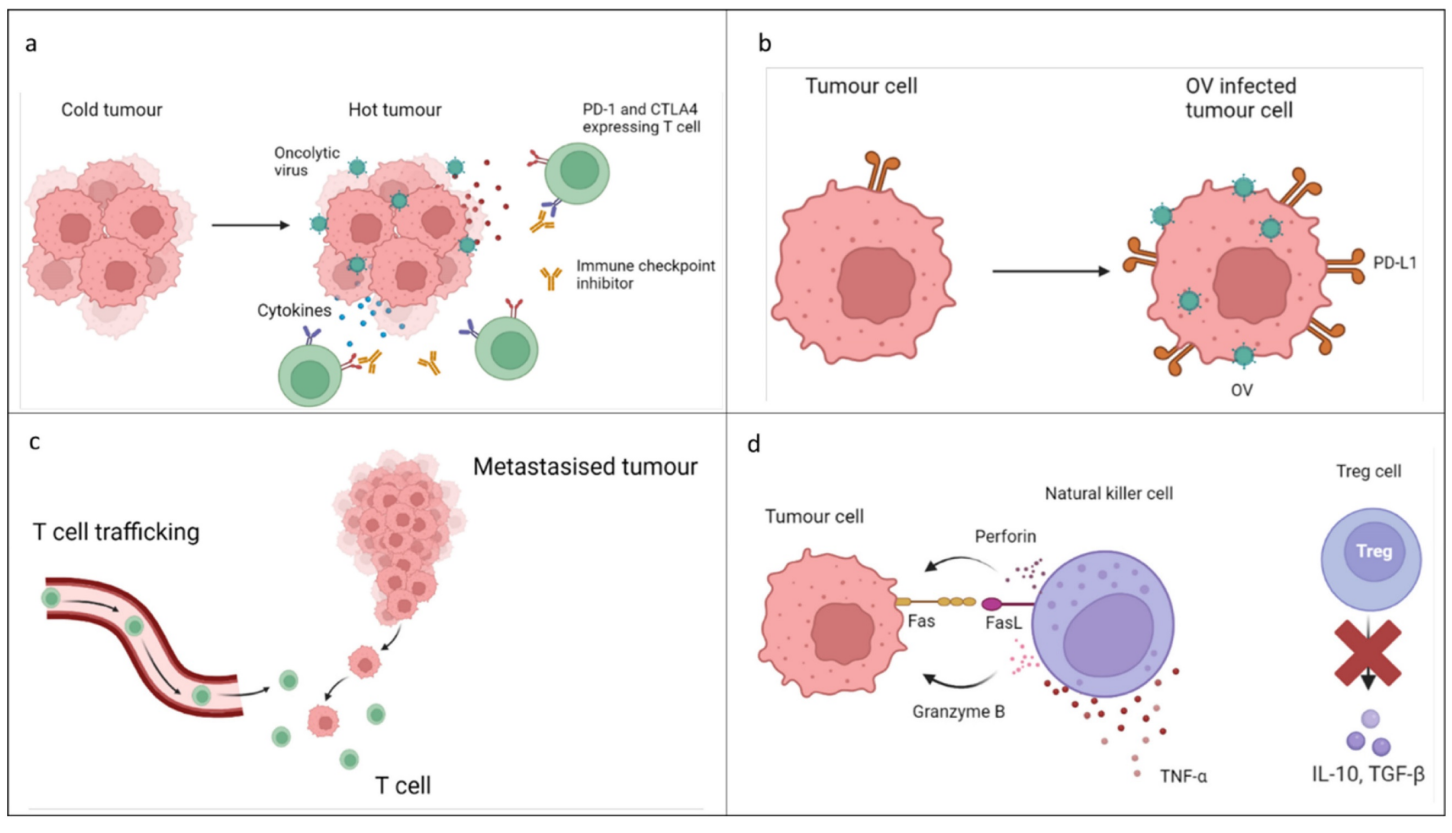
** a.** Alteration of tumour immune microenvironment: OVs (OV) cause an increase in the production of pro-inflammatory cytokines reducing the immune-suppressant tumour microenvironment. This aids in the conversion of tumours into “hot” tumours increasing T cell infiltration and allowing immune checkpoint inhibitors (ICI) to work more effectively. **b.** Increase in PD-L1 expression: Although the mechanism is currently undescribed, combination of OV with ICI has been associated with increased interferon gamma release resulting in upregulation of PD-L1 expression leading to higher efficacy of anti PD-1 therapy. **c.** Increase in CD8+ T cell infiltration: Combination therapy has shown an increase in systemic anti- tumour response in distant metastasised tumours by an increase in trafficking of CD8+ T cells. **d.** Increase in natural killer cell activity and decrease in regulatory T cell activity: Natural killer cells are more likely to kill OV infected cells and anti PD-1 treatment resulted in an increase in tumour necrosis factor α. Combination therapy results in a reduction in regulatory T cell activity creating a more favourable tumour microenvironment for ICI.^5^, ^14^, ^27^,^29^

Lastly, an important innovation in OVT is the application of mesenchymal stem cells to deliver the OV to the tumour where the cells exhibit tumour tropic migration (Figure 2). 75

### Comparison of OVs, immunotherapy, chemotherapy and radiotherapy

### Assessing clinical outcomes

There are several possible endpoints in terms of efficacy of OVT and the overall survival rate, including ORR and clinical benefit rate (CBR) are considered as well as duration of response (DoR) and duration of clinical response (DoCR). Factors also considered to calculate the objective response and treatment outcomes include the treatments' complete response, (CP) partial response (PR), and stable or progressive disease (SD, PD). 76

### Future perspectives

Some clinical trials could explore the different combinations of chemotherapy drugs, with ICI and a type of CAR-T-cell with one or more different OVs against the same cancer type. This approach could be undertaken to clarify the efficacy of different combinations.

Further, the development of combination therapy with CART-cells and OVs may improve efficacy in solid tumours, possibly using multi-specific T-cell engagers and nanoengineering with liposomes and cell-penetrating proteins. 77

Also, gene therapy is possible using engineered OV as a vector for DNA or RNA delivery to alter the TIME by changing the cytokine dynamics of the tumour and enhancing the host immunogenicity of the tumour, making it 'hot'.^49^ An early example of this approach is an OV, LOAd703 combined with chemotherapy for unresectable metastatic pancreatic cancer that is found to be a safe and feasible therapy. New combinations of chemotherapy antimicrotubular agents like Paclitaxel and ICI antibodies, like Atezolizumab or pembrolizumab (PD-1 inhibitor), are being considered to extend this approach. 78-80

OVs can be combined with antibody therapy in trans fashion, and the outcome can be well predicted whereas viruses encoded with antibodies as 'cis' agents, by genetic engineering, may enhance OT delivery to its target, directing the therapy accurately where it is needed. This genetic approach can reduce systemic antibody toxicity, and enhance the delivery of multiple biologics to the tumour. 81 Is there a limit to the number of transfected elements that can be inserted into OVs? Do they all work effectively in combination or interact negatively with each other? This all needs to be evaluated when translating preclinical trials to the clinical environment, where humans can often respond very differently to animal disease models.

While TIME-altering therapies hold promise as therapeutic targets, reversing the epigenetic alterations and posttranslational ubiquitination of proteins that occur in tumorigenesis are promising new approaches to cancer drugs as the understanding of oncogenesis deepens. Several approaches to this are being trailed and their inclusion with OVT as the genetic vectors may advance these treatments. CD 73 is an immune checkpoint enzyme in cancer that promotes tumour aggression by suppressing the recruitment of leukocytes to the tumour but can be stabilised by the deubiquitinating enzyme OTUD4. 10,82,83 Further, understanding of the cross-play between acetalization and ubiquitination and their role in anti-tumour attack may be a key factor in adapting OVTs by enhancing the host tumour immune responses. 78

Despite the promise of immunotherapy to treat cancer, it is clear that TIME can limit the efficacy of some therapies. However, some combination therapies with demethylating agents have been shown to enhance the TIME for pyro-proptosis, due to cellular viral infection. A recent example of a strategy to improve the immunogenicity of a tumour is by combining a DNA methyltransferase inhibitor antibody conjugated nanoprodrug, of the epigenetic inhibitor, 5-Azacytidine, with oncolytic HSV. 81,84

Enhancing the immune lymphocyte recruitment and activation against tumours, by transfecting OVs with deubiquitinating enzyme genes and reversing the anti-inflammatory effects of CD73, may be novel ways of improving the current outcomes of OVTs. Further understanding of the cross-play between acetalization and ubiquitination and their role in anti-tumour responses may be important in adapting OVTs by enhancing the host tumour immune responses. 83 Alternatively, combining anti-CD73 antibodies with OVs may improve treatment outcomes. 82,85 Could the inhibition of CD73 be feasible with an OVT in a cis or trans approach to enhance host immune responses to a tumour, thus turning it from 'turning up the heat' from 'cold to hot'?

Other possible enhancements in the design for genetically engineering OVs as viral vectors include genes for enzymes that disrupt the tumour extracellular matrix and factors for manipulating metabolic enzymes to reverse metabolic programming. In addition to these strategies, OVs can be designed to express antiangiogenetic elements that inhibit angiogenesis and the supply of nutrients and oxygen to the tumour. However, in a large solid tumour, the resulting central necrosis may be difficult to manage. 6

As only a portion of cell receptors are known for half of the 200 viruses recognised, a full understanding of this area and knowledge of the specific tropism possible for an oncovirus in a human patient is an essential research target to enable this valuable resource to be optimised. 86

As some cancers lack successful treatments, the specific focus could develop innovative therapy strategies for these cancers using some of these new combinations of drugs. Matching target malignant tissue to appropriate viruses is an important element of research, enhancing the tropism of the tumour lysis. The development of mesenchymal cells could enhance the tropic delivery of OVs, as they can be bioengineered to enhance cancer therapy delivery to the appropriate target. Currently, clinical trials are evaluating this area of treatment for cancer patients. 87

A rubric cube analogy of several different combined strategies of multiple elements may provide an exciting future for cancer treatments with patient-centred genotype and phenotype-specific treatments. The use of AI to design new personalised approaches to these combinations for cancer patients may be a valuable tool for future cancer therapies. Also, the translation of successful immunotherapies from one type of cancer to treat another condition by repurposing their use in new clinical trials will expand the current treatment boundaries for cancer treatment.

Therapeutic interventions into cancer therapies remain a challenging focus for the future and strategies to combat cancer by overcoming resistance mechanisms to current treatments, whilst translating the recent successes in animal models to human cancer patients continue to motivate researchers in this valuable area of scientific discovery.

## Conclusion

OV combination therapies have resulted in variable levels of success. The combinations of OVT with different chemotherapy drugs produced positive results relating to safety with few adverse events. However, the studies and specific combinations discussed did not always lead to a significant difference in efficacy, when compared to monotreatment. The combination with ICI proved more successful when OVT was combined with ipilimumab as opposed to pembrolizumab. Combination with ipilimumab could be a beneficial method to deliver ICI treatment to a wider range of patients as they are currently only successful in a small subset of patients. Adverse events for ICI and OVT combination were lower than therapy with multiple ICI drugs suggesting OVT is not only a more successful treatment, but also a safer alternative. Phase III trials for T-VEC in combination with ipilimumab using double-blinded, randomised, placebo-based controls should be the next step in testing their effectiveness. Finally, initial laboratory-based *in vitro*, and *in vivo* experiments were extremely promising for combining OVT with CAR-T-cell therapy with mice survival being significantly improved. However, many translational challenges exist in translating animal models into clinical successes.^68^

Moving these experiments into the next stage such as non-human primate testing and human testing for safety and efficacy trials would be hugely encouraging for the future of novel oncolytic viral cancer treatments. However, the number of possible combinations of different drugs, viruses and cancers is extensive therefore future research should focus on using the FDA-approved T-VEC virus in combination with ICI against cancers with the lowest survival rates and that lack treatments such as pancreatic cancer.

## Author contributions

Emily Charlotte- first draft of the article.

Dr Annwyne Houldsworth-the conception and design of the study, writing, revising and editing, final approval of version to be submitted.

## Figures and Tables

**Table 1:** Five ongoing or currently recruiting studies combining oncolytic viral therapy with either chemotherapy, immune checkpoint inhibitors or CAR-T-cell therapy for a range of cancers. 29-33

Summary of ongoing and recruiting oncolytic virus combination studies			
Therapy combination	Cancer type	Trial type	Trial identification number
T-VEC + paclitaxel	Breast cancer, ductal carcinoma	Phase II Single arm	NCT02779855
ASP9801 OV + pembrolizumab	Advanced metastatic solid tumours	Phase I Non-randomised	NCT03954067
Adenovirus + HER2 specific CAR-T-cell	HER2 positive cancers	Phase I Single arm	NCT03740256
T-VEC + ipilimumab, nivolumab	Triple-negative oestrogen receptor positive, HER2 negative breast cancer	Phase I Single arm	NCT04185311
Olvi-Vec + platinum-doublet + bevacizumab	Ovarian cancer	Phase III Randomised control trial	NCT05281471
CF33-hNIS-antiPDL1 Chimeric orthopox virus	Metastatic triple negative breast cancer	Phase I Evaluating safety, side effects and best dose	NCT05081492

**Table 2:** An overview of some of the cancer treatment concepts comparing and contrasting their modes of action, disadvantages and advantages. 15, 18, 19, 24, 29, 51, 71, 76

Main elements	Applications	Mode of action	Specific advantages	Disadvantages	Delivery	Hot/cold
T-VEC oncolytic virus	Targeted oncolytic virus to tumour, infects cancer cells and destroys them, can also be viral vector	Host immune recruitment, cell lysis by the virus	Targeted, effective with mild side effects, promotes immune response	Host immune response to virus, delivery of therapy	IV, local intratumoral	Cold to hot
Immune checkpoint inhibitors	Blocks PD-1, PD-L1, CTLA-4, enabling tumour cell apoptosis	Apoptosis of tumour cells	Enhanced apoptosis of tumour cells	Overactive immune system leading to inflammation, complications and side effects	Intravenous	Cold to hot
Viral delivery of cytokine mimics and inhibitors	IL12, IL2, IFN, TNF, growth factors, binding proteins trigger severe immune responses, low tropism to some cells, insertion into host can cause mutagenesis	TIME-enhancing immune response to tumour, decreases tumour proliferation, motility and increases MHC I and antigen presentation	Tumour selectivity, lytic activity, viral infection can activate PAMPS, enhance APCs	Tumour penetration limited, host immune response can neutralize virus, low efficacy when used on their own, tumour cells can become resistant, OVs can revert to their pathogenic state	Local intravenous	Cold to hot
CAR-T cell	Genetic modification of T cells to locate and destroy cancer cells, used to treat leukaemia, lymphoma, multiple myeloma	Immunotherapy with chimeric antigen receptor, TCRs bind to cancer cells, promotes an immune response to tumour	Specific genetic engineering of TCR can target different cancers, sustained remission, better quality of life	Cytokine release syndrome, neurotoxicity, blood disorders, relapses, initial treatment failure, tumour immune escape, poor penetration into solid tumours	Intravenous	Cold to hot
Monoclonal antibodies	Tumour targeting, Ab drug conjugates, immune checkpoint inhibition	Inhibition of receptors that promote tumour proliferation	Targeted activation of immune system	Allergic reactions, capillary leaks, cytokine release syndrome, heart, lung, skin problems, internal bleeding	Intravenous	Cold to hot
Bispecific monoclonal antibodies BsAbs	Blind both tumour cell and cytotoxic cell	Ab for tumour targeting and host immune cell recruitment and host immune attack on tumour	Immune cell redirection, specificity of target to cancer cells	Infections, low white blood cell counts, cytokine release syndrome	Subcutaneous Modified OVs encoding Abs Nanoconstructs	Cold to hot
Systemic chemotherapy	Disrupts cell cycle of cancer cells	Interferes with RNA/DNA synthesis	Prevents spread of cancer, shrinks size of tumour	Cancer and healthy cells targeted, severe side effects, can harm a foetus	Intravenously Oral	Can be given to cold or hot tumours
Radiotherapy	Use of radiation to kill cancer cells or slow their growth	Targets tumour, modulates TIME	Fewer side effects compared to chemotherapy, can shrink tumour before surgery	Healthy tissue can be damaged surrounding the tumour site	External beam Brachytherapy Radioscope Intrabeam	Can be used to treat both cold and hot tumours
